# Germinal center formation is resilient to CD69 deletion on T follicular helper cells

**DOI:** 10.1111/imcb.70051

**Published:** 2025-08-04

**Authors:** Stephane M Guillaume, Helena A Carslaw, Silvia Innocentin, Louise M C Webb, Adrian Liston, William S Foster, Michelle A Linterman

**Affiliations:** ^1^ Immunology Program Babraham Institute Cambridge UK; ^2^ Department of Pathology University of Cambridge Cambridge UK; ^3^ Present address: Department of Pathology, NYU Langone Grossman School of Medicine New York University New York NY USA

**Keywords:** CD69, follicular helper T‐cell, germinal center, immunization

## Abstract

T follicular helper (T_FH_) cells are a helper T‐cell subset that is defined by their localisation to B‐cell areas of secondary lymphoid tissues, enabling them to provide their B‐cell helper function. Precursors of T_FH_ cells migrate to the B‐cell follicles by upregulating CXCR5 and downregulating CCR7, a process that can be blocked by S1PR1 overexpression. T_FH_ cells and their precursors also express the early activation antigen CD69, which is a negative regulator of S1PR1. In this study, we tested the hypothesis that CD69 expression by T_FH_ cells is important for their differentiation and localisation after immunization. Genetic deletion of CD69 on T_FH_ cells and a proportion of their precursors did not alter their formation, nor their ability to support high‐affinity B‐cell responses. This demonstrates that although CD69 is expressed highly on T_FH_ cells, it is not necessary for their formation or their B‐cell helper functions in lymph nodes (LNs).

## Graphical abstract

CD69 is upregulated on human and mouse CD4^+^ T follicular helper (T_FH_) cells within the germinal center. In this article, we use an *Il21*‐cre mouse strain to ablate floxed *Cd69* genes from T_FH_ cells and a proportion of their precursors. We find that when T_FH_ cells lack CD69, the germinal center response remains intact, supporting B‐cell affinity maturation and the generation of memory B cells (MBCs) and plasma cells at normal frequencies. Following secondary immunization, the germinal center response remained unperturbed, demonstrating that the germinal center is resilient to deletion of CD69 from T_FH_ cells.

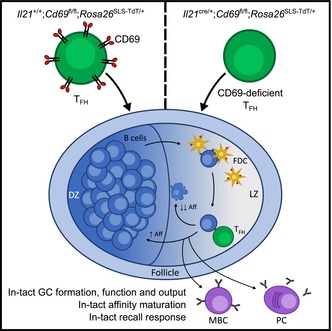

## INTRODUCTION

The early activation antigen CD69 is expressed by many different types of immune cells and has been reported to have several distinct roles in the immune system. Also known as activation inducer molecule (AIM) or C‐type lectin domain family 2 member C (CLEC‐2C), CD69 is a membrane‐bound homodimeric receptor found on granulocytes, monocytes, macrophages,^1^ platelets,^2^ NK cells,^3^ T cells, and B cells.^4^ On lymphocytes, CD69 is one of the earliest cell surface proteins to be upregulated upon cell activation, appearing within 2–3 h of stimulation.^5^ On T cells, CD69 has primarily been studied in the context of tissue‐resident memory T cells (T_RM_), where a link has been drawn between CD69 expression and tissue retention^6^, ^7^, ^8^ which has been ascribed to the role of CD69 as a negative regulator of sphingosine‐1‐phosphate receptor 1 (S1PR1).^8^, ^9^ The CD69‐S1PR1 interaction prevents S1P‐mediated migration of lymphocytes to the blood and efferent lymphatics. In addition to its proposed role in tissue retention,^8^ CD69 has also been implicated in T‐cell IL‐22 secretion^4^, ^10^ and may have a role in regulatory T‐cell (T_REG_) and T‐helper 17 (T_H_17) differentiation.^11^, ^12^, ^13^

CD69 has been shown to be highly expressed on T follicular helper (T_FH_) cells,^7^, ^14^, ^15^, ^16^ but its function on this subset has not been established. T_FH_ cells have key roles in humoral immunity, where they localize to B‐cell areas of lymphoid tissues and support B‐cell proliferation, class‐switch recombination, and the longevity of the germinal center (GC) reaction.^17^ Localization of T_FH_ cells to, and retention within, B‐cell regions of lymphoid tissues requires increased expression of CXCR5 and S1PR2, alongside decreased expression of CCR7 and S1PR1.^18^, ^19^, ^20^ Given that T_FH_ cells in both mice and humans have been reported to express high levels of CD69^7^, ^14^, ^15^, ^16^ this study aimed to determine whether CD69 has a role in T_FH_‐cell localization or function after immunization.

To test this hypothesis, we generated mice whose *Il21*‐producing cells and their progeny lacked functional CD69 protein (*Il21* ^cre/+^;*Rosa26* ^LSL‐TdTomato^;*Cd69* ^fl/fl^),^21^ and tested their immune response to protein subunit vaccination. The absence of CD69 on *Il21‐*fatemapped cells did not alter the numbers of T_FH_ cells within GCs, nor the formation or output of the response following vaccination. Consistent with an intact GC response, there was comparable generation of memory B‐cell (MBC) and plasma‐cell (PC) populations and intact affinity maturation of serum antibodies. The recall response to a second homologous immunization was likewise intact, indicating that loss of CD69 from *Il21*‐fatemapped cells does not impact B‐cell recall responses. This provides evidence that CD69 expression on T_FH_ cells is dispensable for GC size and output in response to protein‐adjuvant vaccination.

## RESULTS

### Heightened expression of CD69 on T_FH_ cells in humans and mice

**Figure 1:**
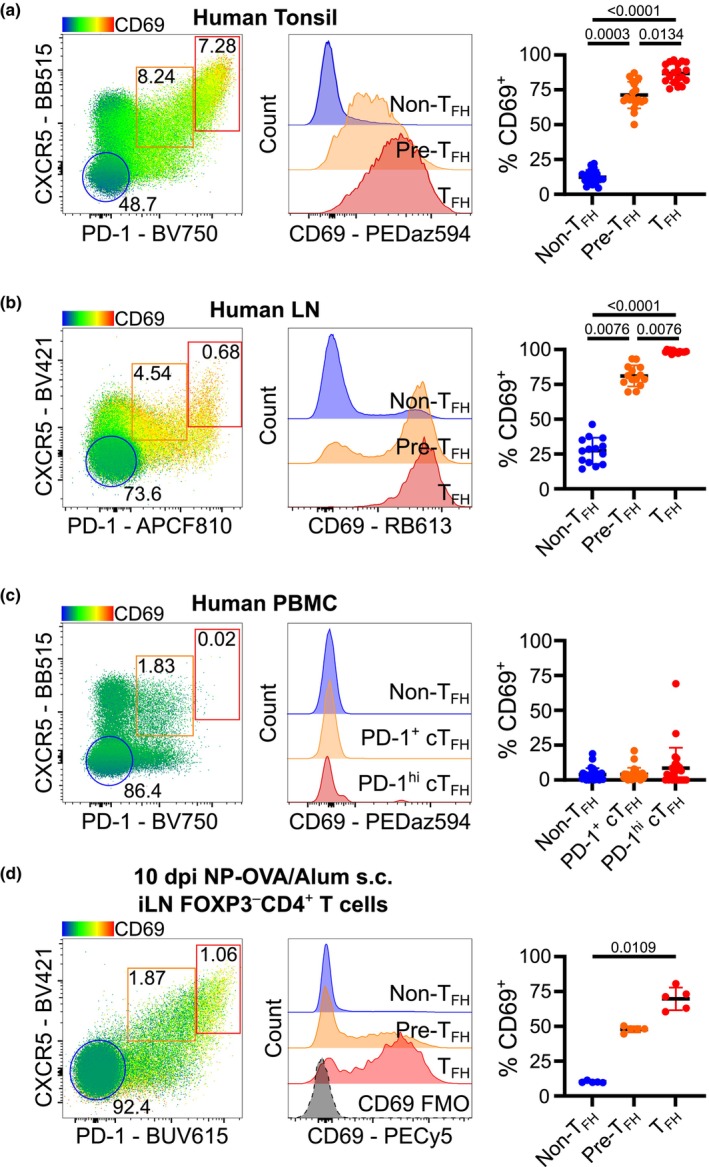
Human and mouse lymphoid tissue T_FH_ cells are enriched for CD69 expression. **(a–d)** CD69 expression flow cytometry heatmap of CD4^+^ T_CONV_ (FoxP3^−^) cells, CD69 expression histogram, and frequency of T‐cell populations expressing CD69 for **(a)** human tonsil (*n* = 19), **(b)** human iliac lymph node (*n* = 14), **(c)** human peripheral blood mononuclear cells (*n* = 26), and **(d)** mouse iLN 10 days following subcutaneous NP‐OVA/Alum immunization. **(a–c)** Data are pooled from multiple experiments. **(d)** Data are representative of two independent experiments with five mice per group. **(a–d)** Contour plots and histograms are representative samples. Numbers in contour plots represent percentage population gated of parent population. Symbols on graphs indicate independent samples; lines represent mean, and error bars indicate s.d. Statistics used are Mann–Whitney tests with Holm‐Šídák multiple corrections.

CD69 has been previously reported to be highly expressed on T_FH_ cells from both mice and humans^20^, ^22^, ^23^ which is thought to reflect their constant T‐cell receptor triggering during cognate interactions with B cells.^24^ We confirmed high expression of CD69 on the surface of human tonsil and lymph node (LN) CXCR5^high^PD‐1^high^ T_FH_ cells compared to PD‐1^low^CXCR5^−^ non‐T_FH_ cells, with intermediate expression on cells with a T_FH_‐precursor CXCR5^+^PD‐1^intermediate^ (pre‐T_FH_) phenotype (Supplementary figure 1a, Figure 1a, b). High expression of CD69 was a feature of T_FH_ cells in secondary lymphoid tissues, which was not observed in the CXCR5^+^PD‐1^+^ circulating counterpart of T_FH_ cells (Supplementary figure 1b, Figure 1c), consistent with previous literature demonstrating higher expression of S1PR1 on circulating T_FH_ cells (cT_FH_) in human blood compared to LNs.^25^ To validate CD69 expression on mouse T_FH_ cells, mice were immunized subcutaneously (s.c.) with the hapten 4‐hydroxy‐3‐nitrophenyl acetyl coupled to ovalbumin (NP‐OVA) mixed with Alum adjuvant in the left flank, and the inguinal LN (iLN) was analyzed 10 days postimmunization (dpi). Analysis of FoxP3^−^CD4^+^ T cells from the iLN during the GC response indicated ~10% of PD‐1^low^CXCR5^−^ non‐T_FH_ cells express CD69 compared to > 60% of CXCR5^high^PD‐1^high^ T_FH_ cells (Figure 1d). This confirms that T_FH_ cells express CD69, with a similar expression pattern in both mice and humans.

### CD69‐deficient *Il21*‐fatemapped cells localize to GCs

One of the identified roles of CD69 for T cells includes the sequestration of S1PR1, reducing the S1P‐mediated emigration of T_RM_ cells from peripheral and lymphoid tissues to the circulation.^8^, ^9^ To test the hypothesis that CD69 is important for the retention of T_FH_ cells in the GC, we developed and characterized the *Il21* ^cre/+^;*Rosa26* ^LSL‐TdTomato^;*Cd69* ^fl/fl^ mouse line in which all *Il21*‐expressing cells have cre recombinase activity. In *Il21* ^cre/+^;*Rosa26* ^LSL‐TdTomato^;*Cd69* ^fl/fl^ mice, *Il21*‐expressing cells and their progeny should simultaneously excise exons 2, 3, and 4 of the CD69 gene^26^ and excise the stop codon downstream of the *Rosa26* (*R26*) promoter, resulting in the constitutive production of TdTomato (TdT). We have previously shown that the *Il21* ^cre^ mouse strain fate maps the majority of T_FH_ cells, as well as ~50% of CXCR5^+^PD‐1^intermediate^ pre‐T_FH_ cells.^21^

**Figure 2:**
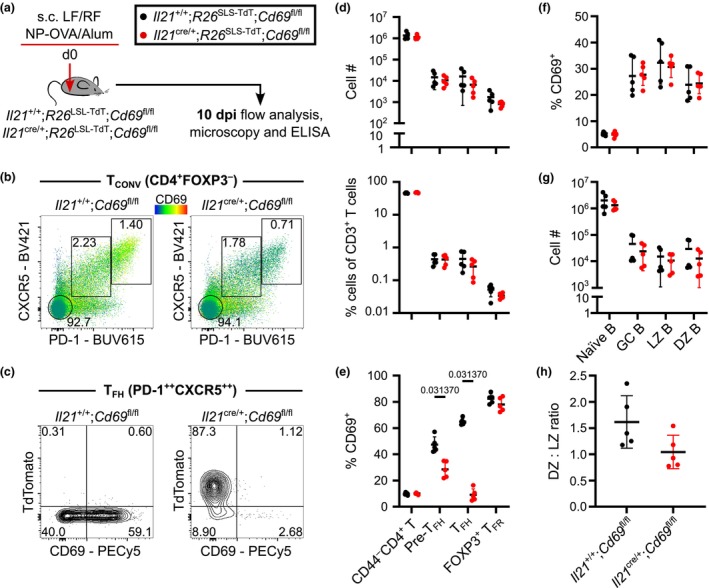
TdTomato^+^ *Il21*‐expressing cells are deficient for CD69. **(a)** *Il21* ^+/+^;*R26* ^LSL‐TdT^;*Cd69* ^fl/fl^ or *Il21* ^cre/+^;*R26* ^LSL‐TdT^;*Cd69* ^fl/fl^ mice received NP‐OVA/Alum s.c. in LF and RF for day 10 flow cytometry, microscopy, and ELISA analyses. **(b)** CD69 expression flow cytometry heatmap of 10 dpi CD4^+^FoxP3^−^ T_CONV_ cells on *Il21* ^+/+^;*R26* ^LSL‐TdT^;*Cd69* ^fl/fl^ and *Il21* ^cre/+^;*R26* ^LSL‐TdT^;*Cd69* ^fl/fl^ iLN samples. **(c)** PD‐1^++^CXCR5^++^ T_FH_ contour plots on *Il21* ^+/+^;*R26* ^LSL‐TdT^;*Cd69* ^fl/fl^ and *Il21* ^cre/+^;*R26* ^LSL‐TdT^;*Cd69* ^fl/fl^ iLN samples. **(d)** Enumeration and frequency of CD4^+^ T‐cell populations. **(e)** Frequency of CD4^+^ T‐cell populations expressing CD69. **(f)** Frequency of B‐cell populations expressing CD69. **(g)** Enumeration of B‐cell populations. **(h)** Quantification of DZ:LZ ratio. **(b, c)** Data are representative of two independent experiments with five mice per group. Numbers in contour plots represent the frequency of the parent population gated. **(d–h)** Data are representative of two independent experiments with a total of five mice per group. Symbols on graphs indicate individual mice; lines represent the mean; error bars indicate s.d. Statistics used are Mann–Whitney tests with Holm‐Šídák multiple corrections.

Both control *Il21* ^+/+^;*R26* ^LSL‐TdT^;*Cd69* ^fl/fl^ and *Il21* ^cre/+^;*R26* ^LSL‐TdT^;*Cd69* ^fl/fl^ experimental mice were immunized s.c. into both left and right flanks (LF and RF) with NP‐OVA/Alum and were culled at 10 dpi for analysis by flow cytometry, microscopy, and enzyme‐linked immunosorbent assay (ELISA) to interrogate the early response to immunization (Figure 2a). Flow cytometric analysis of FoxP3^−^CD4^+^ T cells showed that the majority of CXCR5^+^PD‐1^high^ T_FH_ cells had lost CD69 expression, and the few that remained were TdTomato‐negative, indicating they did not have an expression history of the *Il21* ^cre^ allele (Supplementary figure 2a, b, Figure 2b, c). CD69 deletion on *Il21‐*fatemapped cells had no significant impact on the cell numbers nor frequencies of CD44^−^CD4^+^ naïve cells, CXCR5^+^PD‐1^+^ pre‐T_FH_ cells, CXCR5^++^PD‐1^++^ T_FH_ cells, or Foxp3^+^CXCR5^+^PD‐1^+^ T_FR_ cells (Figure 2d). Enumeration of CD69 on these T‐cell subsets confirmed that *Il21* ^cre/+^;*R26* ^LSL‐TdT^;*Cd69* ^fl/fl^ mice successfully delete CD69 on T_FH_ cells (Figure 2e). We interrogated whether CD69 deletion altered the expression of the activation protein inducible T‐cell costimulatory (ICOS) and migratory proteins CXCR5 and CCR7, and found no significant difference between the two groups on any CD4^+^ T‐cell population (Supplementary figure 3a, b). As interaction between CD69 and S1PR1 can facilitate tissue‐retention,^8^, ^9^ we hypothesized that T_FH_ cells lacking CD69 may be more likely to recirculate in the blood. There was no difference in the propensity for PD‐1^+^ T_CONV_ cells to recirculate in the blood, and no change to the amounts of T_FH_ cells isolated from the spleen between the two groups (Supplementary figure 3c, d).

CD69 deletion was found to not affect naïve or GC B‐cell populations (Figure 2f), demonstrating that *Il21* ^cre^‐mediated deletion of CD69 specifically resulted in a T_FH_‐cell population that largely lacked CD69 but formed in numbers comparable to control animals. Consistent with this, the number of KI67^+^GL7^+^ GC B cells and their CXCR4^−^CD86^+^ light zone (LZ) and CXCR4^+^CD86^−^ dark zone (DZ) phenotype was comparable in control and experimental animals (Figure 2g, h).

**Figure 3:**
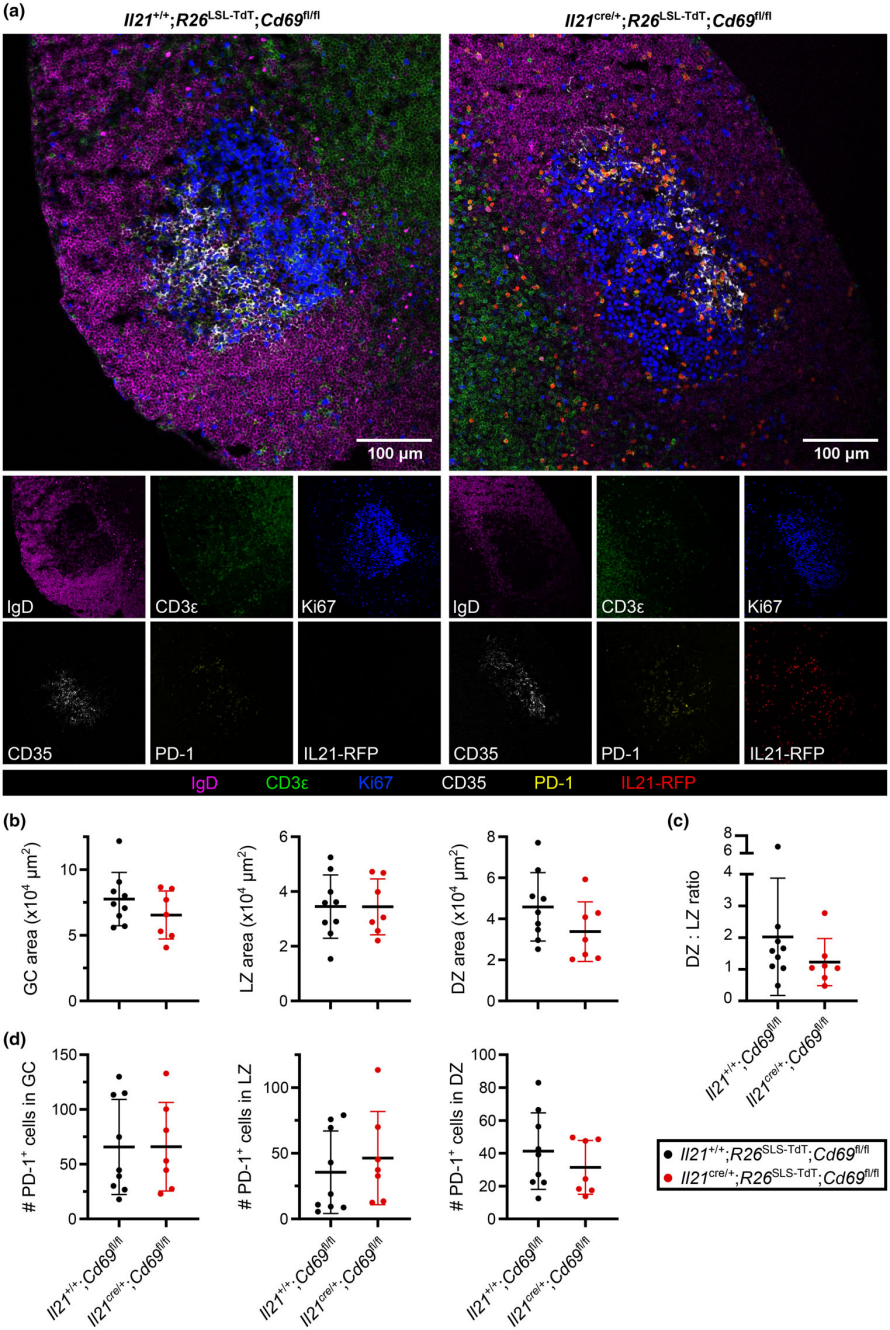
CD69‐deficient *Il21‐*fatemapped cells can support GC structure. **(a)** Representative confocal images of iLN GC regions for *Il21* ^+/+^;*R26* ^LSL‐TdT^;*Cd69* ^fl/fl^ or *Il21* ^cre/+^;*R26* ^LSL‐TdT^;*Cd69* ^fl/fl^, at 20x magnification, 10 days following NP‐OVA/Alum immunization. Below are single‐color images of the same sections. Markers identified in sections include IgD (magenta), CD3ε (green), Ki67 (blue), CD35 (white), PD‐1 (yellow) and IL21‐TdTomato (red, for applicable samples). GCs were identified by expression of Ki67 and CD35, and the LZ was defined by expression of CD35, demarking the FDC network. The DZ was calculated as the GC area without CD35. The scale bar is 100 μm. **(b)** Average GC, LZ and DZ area sizes per LN. **(c)** Quantification of average DZ:LZ ratio per LN. **(d)** Average number of PD‐1^+^ T_FH_ cells in the GC, LZ, and DZ per LN. **(a)** Images are representative of two experiments with two to five LNs per group. **(b–d)** Data are pooled from two experiments with two to five mice per group. Symbols represent individual mice, lines indicate the mean, error bars indicate s.d. Statistics used are Mann–Whitney tests.

To determine whether loss of CD69 on T_FH_ cells resulted in fewer T_FH_ cells within the GC, we quantified T_FH_ cells *in situ* in GCs by confocal imaging. Both control *Il21* ^+/+^;*R26* ^LSL‐TdT^;*Cd69* ^fl/fl^ and *Il21* ^cre/+^;*R26* ^LSL‐TdT^;*Cd69* ^fl/fl^ experimental groups had visible GCs (Figure 3a). In *Il21* ^cre/+^;*R26* ^LSL‐TdT^;*Cd69* ^fl/fl^ mice, TdTomato coexpressed with cells positive for the T_FH_ marker PD‐1, and no TdT^+^ cells were detected in the *Il21* ^+/+^;*R26* ^LSL‐TdT^;*Cd69* ^fl/fl^ samples (Figure 3a). Quantification of GCs showed that there was no difference in the total GC area, nor any significant difference in the LZ or DZ areas, nor their ratio (Figure 3b, c). PD‐1^+^ cell number was unchanged in the absence of CD69 expression when measured in the whole GC as well as in the LZ and DZs (Figure 3d). Collectively, confocal microscopy corroborated the flow cytometric data, confirming no differences to GC size, structure, or T‐cell abundance when CD69 was not expressed by T_FH_ cells.

### CD69‐deficient T_FH_ cells can support affinity maturation

**Figure 4:**
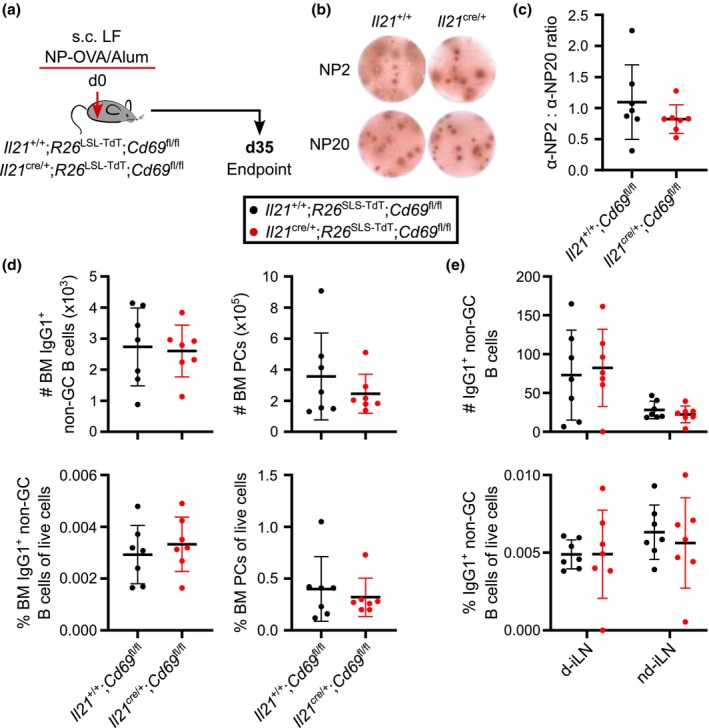
CD69‐deficient *Il21*‐fatemapped cells support antibody‐secreting cell formation and affinity maturation. **(a)** *Il21* ^+/+^;*R26* ^LSL‐TdT^;*Cd69* ^fl/fl^ and *Il21* ^cre/+^;*R26* ^LSL‐TdT^;*Cd69* ^fl/fl^ mice received NP‐OVA/Alum s.c. in LF for day 35 flow cytometry, ELISA, and ELISpot analyses. **(b)** Representative images of α‐NP2 and α‐NP20 IgG1 ELISpot wells. **(c)** α‐NP2: α‐NP20 IgG1 antibody‐secreting cell ratio in the BM. **(d)** Enumeration and frequency of IgG1^+^ non‐GC B cells and PCs present in the BM. **(e)** Number and frequency of IgG1^+^ non‐GC B cells in the draining and nondraining iLNs. **(b)** Images are from two representative samples. **(c–e)** Data are representative of two independent experiments with 7 mice per group. Symbols represent individual mice; lines indicate the mean, and error bars indicate s.d. **(c, d)** Statistics used are Mann–Whitney tests. **(e)** Statistics used are Mann–Whitney tests with Holm–Šídák multiple corrections.

As CD69‐deficient T_FH_ cells could support GC reactions, we next tested whether the output of the GC or quality of the affinity of antibody secreting cells was affected. *Il21* ^+/+^;*R26* ^LSL‐TdT^;*Cd69* ^fl/fl^ and *Il21* ^cre/+^;*R26* ^LSL‐TdT^;*Cd69* ^fl/fl^ mice were immunized s.c. with NP‐OVA/Alum in the left flank (Figure 4a), and MBCs and PCs were assessed 5 weeks later. ELISpot assessment of antigen‐specific antibody secreting cells in the bone marrow showed no difference in the total number nor in the ratio of high to low affinity cells, indicative of intact affinity maturation when T_FH_ cells lack CD69 (Figure 4b, c). Flow cytometric analysis further revealed the number of IgG1^+^ MBCs and PCs in the bone marrow was comparable between the two groups (Figure 4d), as was the number of IgG1^+^ MBCs in the draining and nondraining iLNs (Figure 4e).

### CD69‐deficient T_FH_ cells support MBC formation for recall response

**Figure 5:**
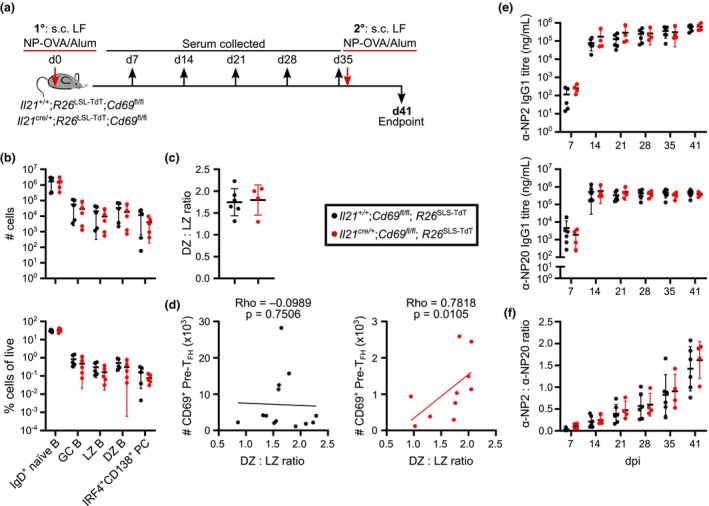
Response kinetics are preserved during secondary immunization when Il21‐fatemapped cells lack CD69. **(a)** *Il21* ^+/+^;*R26* ^LSL‐TdT^;*Cd69* ^fl/fl^ and *Il21* ^cre/+^;*R26* ^LSL‐TdT^;*Cd69* ^fl/fl^ mice were immunized with NP‐OVA/Alum s.c. in LF and received a secondary ipsilateral boost at 35 dpi for 41 dpi flow cytometry, with weekly bleeds for serum ELISA. **(b)** Quantification and frequency of B‐cell and PC populations. **(c)** Enumeration of DZ:LZ ratio. **(d)** Correlation of the number of CD69^+^ pre‐T_FH_ cells and DZ:LZ ratio in the iLN of *Il21* ^+/+^;*Cd69* ^fl/fl^;*R26* ^LSL‐TdT^ (left) and *Il21* ^cre/+^;*Cd69* ^fl/fl^;*R26* ^LSL‐TdT^ (right). **(e)** α‐NP2 and α‐NP20 IgG1 serum antibody titles over time. **(f)** α‐NP2: α‐NP20 IgG1 serum antibody ratio over time. **(b, c, e, f)** Data are representative of two experiments with four to six mice per group. Symbols represent individual mice, lines indicate the mean, error bars indicate s.d. **(d)** Data are pooled from two independent experiments with four to six mice per group. Symbols represent individual mice. Spearman correlations were used to determine the slope and significance. **(b, e, f)** Statistics used are Mann–Whitney tests with Holm–Šídák multiple corrections. **(c)** Statistics used are Mann–Whitney tests.

The primary GC response to NP‐OVA/Alum appeared unaffected by the lack of CD69 expression on T_FH_ cells 10 days after immunization. However, as CD69 deficiency on all T cells has been implicated in poor recall antibody responses^7^, ^24^ we next tested whether a lack of CD69 on T_FH_ cells impacted the response to secondary immunization. Mice were immunized s.c. in the left flank, weekly serum samples were taken, then mice were reimmunized ipsilaterally 5 weeks after the initial immunization (Figure 5a). The number of recall GC B cells and PCs were of a similar magnitude 6 days after secondary immunization in mice whose T_FH_ cells lack CD69 compared to control animals (Figure 5b), with comparable ratios of light and dark zone GC B cells (Figure 5c). Despite no differences in the DZ: LZ ratio between control and experimental mice, there was a significant correlation between the number of CD69‐expressing pre‐T_FH_ cells and the DZ: LZ ratio in *Il21* ^cre/+^;*R26* ^LSL‐TdT^;*Cd69* ^fl/fl^ mice (Figure 5d). In the serum, IgG1 α‐NP2 and α‐NP20 antibody titres increased by over 100‐fold between 7 dpi and 14 dpi in both *Il21* ^+/+^; *R26* ^LSL‐TdT^;*Cd69* ^fl/fl^ and *Il21* ^cre/+^;*R26* ^LSL‐TdT^;*Cd69* ^fl/fl^ groups (Figure 5e). While a steady increase in α‐NP2: α‐NP20 ratio signified an increasing antibody affinity toward NP with time, and an increase in affinity evident postboost (Figure 5f), there was no difference in the titer nor antibody affinity between the two groups at any of the assessed timepoints (Figure 5e, f). These data suggest that affinity maturation mechanisms are functional in the absence of CD69 on T_FH_ cells. Despite CD69 deletion on T_FH_ cells, no measurable impact was detected on GC function by measure of isotype‐switched IgG1^+^ MBC or PC output. Serum antibody titres were unaffected, suggesting that affinity maturation proceeded unhindered in response to both primary and secondary immunization in mice whose T_FH_ cells lacked CD69 compared to *Il21* ^+/+^; *R26* ^LSL‐TdT^;*Cd69* ^fl/fl^ littermates. This intact recall response and subsequent GC formation suggest that the LN GC response can proceed as normal, demonstrating that T_FH_ expression of CD69 is dispensable for GC size and output in response to protein‐adjuvant vaccination.

## DISCUSSION

In this study, an *Il21*‐fatemapping reporter mouse *Il21* ^cre/+^; *R26* ^LSL‐TdT^;*Cd69* ^fl/fl^ facilitated the deletion of CD69 from T_FH_ cells after immunization. Here we show that CD69 was dispensable on T_FH_ cells for the formation of iLN GCs in response to NP‐OVA/Alum immunization. Lack of CD69 on *Il21‐*fatemapped cells had no discernible impact on serum antibody titres or affinity maturation, nor a measurable effect on the GC size or output of CD138^+^IRF4^+^ PCs and IgG1^+^ non‐GC MBCs, indicating that CD69 does not play a role in the retention of T_FH_ cells to LN GCs, or to the output of the response following vaccination.

Differences in CD69 expression were detected between human T_FH_ cells from tonsils and LNs, and mouse LN samples. In mice, while most T_FH_ cells are positive for CD69, there is bimodal expression, whereas human T_FH_‐cell CD69 expression more closely resembled a normal distribution. These differences may reflect inherent differences between mouse and human GC biology. It is also possible that the type of immune stimulus is contributing, as the human tissues sampled in this study were not taken following vaccination. The human samples likely include polyclonal immune reactions involving T_FH_ cells responding to a wider range of antigenic stimuli in different states of activation that may result in the observed heterogeneous and unimodal expression of CD69. In contrast, our protein subunit vaccinated mouse model evaluated T_FH_ cells responding to immunization at discrete time points postvaccination. Investigating CD69 on antigen‐specific T_FH_ cells in human vaccination studies would be ideal as a comparator to the vaccinated mouse model.

While some lymphocyte subsets use CD69 for retention,^8^ a lack of CD69 on T_FH_ cells did not result in fewer T_FH_ cells within GCs. An alternative hypothesis may be that CD69 is upregulated on LN T_FH_ cells primarily due to antigenic stimulation. Its role of sequestering surface expression of S1PR1 may be redundant in the GC, as FDC‐expressed CXCL13 may be a more powerful chemoattractant for T_FH_ cells than S1P, due to its proximity. Alternatively, T_FH_ cells may need CD69 earlier in their differentiation trajectory, consistent with the requirement of S1PR1 downregulation for T_FH_‐cell formation.^20^ A feature of the *Il21* ^cre/+^;*R26* ^LSL‐TdT^;*Cd69* ^fl/fl^ model is that not all pre‐T_FH_ cells lack expression of CD69, which would facilitate T_FH_‐cell formation if CD69 were required at this stage. Overall, this study shows that T_FH_‐cell‐expressed CD69 is dispensable for their localization to GCs, and that the output of GCs is intact. This work supports a model in which CXCR5 and S1PR2 expression are the key migration and retention signals for T_FH_ cells within GCs.

## METHODS

### Human tissue samples

Peripheral blood mononuclear cells were prepared from leucocyte cones, as previously described,^27^ with UK local research ethics committee approval (REC reference 23/YH/0200). Tonsils were sourced from Addenbrookes Hospital general surgery lists. Ethical approval was obtained from the Southeast Coast – Surrey Research Ethics Committee: Study title: *How T follicular helper T cells support B‐cell responses in humans*. REC reference: 16/LO/0453. IRAS project ID: 196604. Human LN^28^ from patients requiring renal replacement therapy. At the time of transplant, lymphoid tissue was removed to allow access to iliac vessels as part of the routine operative procedure. LN samples were collected under ethical approval from UK Health Research Authority, REC references 11/EE/0355 at Cambridge University Hospitals. Written informed consent was received from all volunteers. Samples were collected in accordance with the latest revision of the Declaration of Helsinki and the Guidelines for Good Clinical Practice as described previously.^27^, ^29^

Human samples were processed in a biosafety cabinet within a biosafety laboratory class 2 room. Single cell suspensions were generated and resuspended in fetal bovine serum (FBS) containing 10% DMSO at a concentration of 2 × 10^7^ cells/mL in each cryovial, then frozen using a Biocision CoolCell LX cell freezing container in a −80°C freezer, where they were kept for long‐term storage. When required, samples were thawed as quickly as possible in a 37°C heated metal bead bath, then transferred to RPMI containing 10% FBS and centrifuged. Supernatant was removed and cells were washed twice with PBS. Live cells were quantified, and the sample was resuspended at a concentration of 10^7^ cells/mL. 200 μL was transferred to a 96‐well plate for staining.

### Mice

*Il21* ^+/+^;*R26* ^LSL‐TdT^;*Cd69* ^fl/fl^ and *Il21* ^cre/+^;*R26* ^LSL‐TdT^;*Cd69* ^fl/fl^ mice were generated by crossing *Cd69* ^*fl/fl*^ mice,^26^ *Il21* ^*cre*^ mice^30^ and fatemapping *Rosa26* ^*Lox‐Stop‐Lox‐TdTomato*^ mice,^28^ which were maintained at the Biological Support Unit of the Babraham Institute. No primary pathogens or additional agents listed in the FELASA recommendations were detected during health monitoring surveys of the animal holding rooms.^31^ Ambient temperature was approximately 19–21°C, with a relative humidity of 52%. Light sources operated on a 12‐h light:12‐h dark cycle, flanked by 15 mins of “dawn” and “dusk” periods of subdued lighting. After weaning, mice were transferred to individually ventilated cages with 1–5 mice per cage. Mice were fed calorie restriction mimetics (pelleted) diet (Special Diet Services) *ad libitum* and received seeds (e.g. millet, sunflower) during cage‐cleaning as part of their environmental enrichment. All mouse experimentation was approved by the Animal Welfare and Ethical Review Body at Babraham Institute. All experimental mice were aged between 8 and 16 weeks when commencing experimentation, both male and female mice were used. Animal husbandry and experimentation complied with existing European Union and United Kingdom Home Office legislation (Scientific Procedures Act 1986) and local standards (PPL: P4D4AF812 and PP9973990; PEL: XAE4C054D).

NP‐OVA/alum was administered s.c. to the left and/or right flank of the lower body to induce GC responses in the iLN. NP‐OVA/Alum was prepared by mixing NP‐OVA (1 mg/mL, LGC #N‐5051‐100) with alhydrogel (2%, Invivogen, #vac‐alu‐50) in a 1:1 ratio. Mice were temporarily anesthetized, and a 100 μL bolus injection of NP‐OVA/Alum was administered s.c. per flank. For 10 dpi experiments, mice were immunized in both the LF and RF. For experiments longer than 10 days, mice were immunized on only the left flank. For 10 dpi experiments, the immunized left flank iLN was taken for flow, and the immunized right flank iLN was collected for microscopy. Blood was collected to acquire serum samples for ELISAs. For experiments longer than 10 days, blood samples were acquired every 7 days postimmunization to collect serum for ELISAs. The immunized left flank iLNs and unimmunized right flank iLNs and bone marrow from both hind legs were collected for flow cytometric analysis. The bone marrow was also used for ELISpots.

### Flow cytometry

LNs for flow cytometric analysis were isolated and placed into PBS. Single‐cell suspensions were prepared by mashing organs through a 70 μm nylon filter mesh set within a 6‐well plate using a syringe plunger. Filters were rinsed once with FACS buffer (2% FBS [Sigma Aldrich, #F4135] [v/v] in PBS, 1 mM EDTA [Sigma Aldrich, #6381‐92‐7]), and contents were transferred to 15‐mL tubes. Samples were pelleted by centrifuge and the supernatant removed. LNs were resuspended in 400 μL FACS buffer.

All staining steps described below were performed with as little light exposure as possible to avoid photobleaching of fluorophores. Each fluorophore was titrated with both intracellular and surface staining to determine the ideal dilution and staining conditions prior to use.

Cells were plated into 96‐well V‐bottom plates at a density of approximately 2 × 10^6^ cells/well. Surface antibodies (Table 1) were diluted in 100 μL of FACS buffer per sample to create a master mix. Plated samples were resuspended in 100 μL of surface antibody mix and incubated (1–2 h, 4°C). Cells were then washed as follows: 100 μL of FACS buffer was added to dilute the antibodies and the plate was centrifuged. The supernatant was removed by flicking the plate, and 200 μL of FACS buffer was added to wash samples. Plates were again centrifuged, followed by removal of supernatant, after which cells were ready for fixation. Samples that did not require intracellular or intranuclear staining were acquired immediately. Samples requiring intracellular or intranuclear staining were fixed with 100 μL of the fixation/permeabilization staining kit (Invitrogen, #00‐5123‐43) in the dark (20 min, room temperature [RT]). Following fixation, cells were washed with 100 μL permeabilization buffer (Invitrogen, #00‐8333‐56) and centrifuged. The supernatant was removed, and the cells were washed again with 200 μL of permeabilization buffer. Intracellular antibodies (Table 1) were diluted in 100 μL of permeabilization buffer (Invitrogen, #00‐8333‐56) per sample to create a master mix. Samples were resuspended in the intracellular antibody mix and incubated (overnight, 4°C). The following day, samples were washed twice with permeabilization buffer, then washed once with FACS buffer and resuspended in 180 μL FACS buffer, then acquired immediately. Flow cytometry samples were acquired using an Aurora spectral flow cytometer (Cytek) and data was processed using FlowJo (Tree Star, v10.8.1).

**Table 1:** Fluorescent antibodies and dyes used for flow cytometry.

Target	Clone	Fluorophore	Manufacturer
Mouse samples			
ICOS	C398.4A	BV750	BioLegend
CCR7	4B12	BV785	BioLegend
B220	RA3‐6B2	APC‐Fire810	BioLegend
CD138	281‐2	BV711	BioLegend
CD19	1D3	BUV661	BD Biosciences
CD23	B3B4	BUV496	BD Biosciences
CD3	17A2	SB574	BioLegend
CD4	GK1.5	SV538	BioLegend
CD44	IM7	PerCP	BioLegend
CD69	H1.2F3	PE‐Cy5	BioLegend
CD8a	53‐6.7	BUV805	BD Biosciences
CD86	GL‐1	APC‐Fire750	BioLegend
CXCR4	2B11/CXCR4	BUV563	BD Biosciences
CXCR4	L276F12	BV605	BioLegend
CXCR5	L138D7	BV421	BioLegend
FoxP3	FJK‐16s	APC	Invitrogen
GL7	GL7	PE‐Cy7	BioLegend
IgD	11‐26c.2a	SNIR685	BioLegend
IgG1	A85‐1	BV480	BD Biosciences
IgM	II/41	AF532	ThermoFisher Scientific
IRF4	IRF4.3E4	Pacific Blue	BioLegend
Ki67	16A8	AF700	BioLegend
PD‐1	RMPI‐30	BUV615	BD Biosciences
Streptavidin		AF350	Invitrogen
Viability dye		Viakrome808	Beckman Coulter
Human samples			
CD10	HI10a	BUV737	BD Biosciences
CD19	HIB19	BUV615	BD Biosciences
CD20	2H7	SNIR685	BioLegend
CD25	MEM‐181	SBV515	Bio‐Rad
CD3	SK7	SB550	BioLegend
CD3	UCHT1	SB574	BioLegend
CD4	SK3	BUV496	BD Biosciences
CD4	SK3	cFluor‐BYG750	Cytek
CD69	FN50	PE‐Dazzle594	BioLegend
CD69	FN50	RB613	BD Biosciences
CD8	SK1	APC‐Fire810	BioLegend
CD8	QA18A37	SV538	BioLegend
CXCR5	RF8B2	BB515	BD Biosciences
CXCR5	J252D4	BV421	BioLegend
FoxP3	FJK‐16s	PE‐Cy5.5	Invitrogen
PD‐1	EH12.1	BV750	BD Biosciences
PD‐1	A17188B	APC‐Fire810	BioLegend
Viability dye		Viakrome808	Beckman Coulter

### ELISA

Mouse blood for serum isolation was collected into microtainer tubes. Tubes were centrifuged using a benchtop microcentrifuge (13 000 rpm, 20 min, 20°C), and the serum was stored at −20°C. ELISA plates were coated with 50 μL NP2‐BSA (2 μg/mL, Biosearch Technologies, #N5050L‐100), NP20‐BSA (10 μg/mL, Biosearch Technologies, #N5050G‐100), or anti‐IgG (10 μg/mL, Southern Biotech, #1030‐01) in PBS. Plates were covered with parafilm and stored overnight at 4°C, then washed six times with PBS‐Tw (0.05% Tween‐20 (Sigma Aldrich, #P1379) (v/v) in PBS) using the BioTek 405 LS plate washer. This wash step was repeated following incubation with blocking buffer (2% BSA (Sigma Aldrich, #A7906) (w/v) in PBS‐Tw), sera, and antibodies. A volume of 200 μL of ELISA blocking buffer was added to each well and incubated for 1 h at RT. Serum was thawed and serially diluted (1:3) down the plate at a starting dilution of 1:50, 1:150, 1:450, 1:1350 in ELISA diluent buffer (0.1% BSA (w/v) in PBS‐Tw). An IgG1 isotype standard (Sigma, #M5284) was used, starting at 50 ng/mL, against which antibody titres could be quantified. Plates were incubated for 1 h at RT. Samples were incubated with 50 μL of HRP‐conjugated antimouse IgG1 (Abcam, #ab97240) antibody diluted at 1/8000 in ELISA diluent buffer for an hour at RT. Plates were developed using 50 μL tetramethylbenzidine (TMB) substrate kit (BioLegend, #421101) until a good dynamic range was observed, at which point the reaction was quenched using 50 μL 0.1 M H_2_SO_4_ (Sigma Aldrich, #339741). The PHERAstar FS plate reader (BMG Labtech) was used to measure absorbance at 450 nm.

### ELISpot

Both hind legs were collected from mice into PBS. Following dissection, bone marrow was extracted from the femur and tibia of both hind legs into RPMI‐1640 (Gibco, #21875‐034). Samples were washed and resuspended in red cell lysis buffer for 3 min, then washed with PBS and centrifuged. After washing single cell suspensions, samples were counted using the CASY TT counter (OLS OMNI Life Science) and resuspended at a density of 10^7^ cells/mL for ELISpot and flow cytometry. Each well of a 96‐well MultiScreen HTS HA filter plate (Sigma Aldrich, #MSHAS4510) was incubated with sterile PBS for 30 min, before coating with 50 μL NP2‐BSA (2 μg/mL) or NP20‐BSA (10 μg/mL) and incubating overnight at 4°C. Plates were washed twice with PBS and incubated with 50 μL of complete RPMI‐1640 (1 h, RT). The single‐cell suspension BM samples were resuspended at a concentration of 2 × 10^7^ cells/mL in complete RPMI‐1640. Cells were serially diluted (1:2) down the plate starting from 2 × 10^6^ cells in the first well and incubated overnight (37°C, 5% CO_2_). The cell suspension was flicked out and rinsed with PBS‐Tw five times, followed by PBS three times and dH_2_O three times. Samples were incubated with 50 μL of HRP‐conjugated antimouse IgG1 antibody diluted at 1/8000 in ELISpot diluent buffer. Samples were washed with PBS‐Tw five times and twice with dH_2_O. Plates were developed using 40 μL of AEC (3‐amino‐9‐ethylcarbazole) chromogen substrate kit (Sigma Aldrich, #AEC101‐1KT) according to the manufacturer's instructions until the plate was well developed. To halt the reaction, the substrate was thoroughly washed out by running the plate under tap water. Plates were left to dry in the dark overnight and the ImmunoSpot plate reader (Cellular Technology Ltd.) was used to capture images of each well for manual counting.

### Microscopy

When required, slides were air‐dried under a laminar flow hood for 30 mins at RT. An unbroken hydrophobic circle was drawn around tissue sections using a PAP pen and allowed to dry. Slides were rehydrated by submerging in wash buffer (0.5% Tween‐20 [v/v] in PBS) 3 times for 5 min, and slides were stored in a humidified chamber for the remainder of the staining protocol. Nonspecific binding of antibodies was minimized by incubation of tissues with blocking buffer 2% BSA (Sigma Aldrich, #A7906) (w/v) and 10% normal goat serum (Sigma Aldrich, #566380, [v/v] in PBS) for 60 mins at RT. Slides were washed three times for 5 min using wash buffer, then permeabilized by adding freshly made permeabilization buffer 2% Triton X‐100 (Sigma Aldrich, #T9284, [v/v] in PBS) and incubated for 30 min at RT. Slides were washed three times for 5 min in wash buffer, after which the primary antibody mix, made up of antibodies from Table 2 in confocal antibody diluent (1% BSA [w/v] in wash buffer), was added to each sample and left overnight at 4°C. Slides were washed three times for 5 min with wash buffer and stained with secondary antibodies made up in confocal antibody diluent with 2% normal goat serum for 2 h in the dark at RT. Slides were quickly rinsed twice with wash buffer and once with PBS, then mounted with hydromount aqueous mounting medium (Geneflow, #HS‐106) and covered with a glass coverslip. These were left at RT until dry. Mounted samples were stored at 4°C until acquisition.

**Table 2:** Antibodies and dyes used for immunofluorescent microscopy.

Target	Clone	Fluorophore	Manufacturer
IgD	11‐26c.2a	FITC	BioLegend
CD3e	17A2	AF700	BioLegend
Ki67	SolA15	eFluor450	Invitrogen
CD35	8C12	Biotinylated	eBioscience
PD‐1	RMPI‐30	AF647	BioLegend
Streptavidin		AF750	Invitrogen

Images were acquired using the Stellaris 8 (Leica) confocal microscope. All GCs per LN were imaged, and sequential GC sections were matched up to find the most central GC section, with a minimum of five sections per GC imaged. Channels were collected in separate frames using a 20x/0.75 numerical aperture air lens. Images were compiled using FIJI software (NIH, 1.53 t). Unbiased quantification of acquired images was performed using a CellProfiler (Broad Institute, v4.2.4) pipeline. The GC area was defined as regions that were CD35^+^ and/or Ki67^+^, within which the GC LZ area was identified as being CD35^+^, and the GC DZ as CD35^−^Ki67^+^. T_FH_ cells were defined as PD1^+^ in the GC area, and the presence and colocalisation of different markers within each GC compartment were identified and enumerated using CellProfiler.

### Statistical analysis

All statistical tests were run in GraphPad Prism software (GraphPad, v9.0.0). All data points were included in the analysis except in cases of technical error or ill mice, in which case these points were excluded. Sample sizes per experiment were contingent on the availability of sex‐ and age‐matched mice and littermate controls. Differences between experimental groups were determined using unpaired Mann–Whitney *U* tests with Holm‐Šídák's multiple comparisons test or Kruskal‐Wallis tests with Dunn's multiple comparisons test, where appropriate. A threshold of *P* < 0.05 was used to determine statistical significance, and only significant statistics are shown. All mouse experimental data are from one representative experiment of at least two, presented as the mean ± standard deviation (s.d.). For human samples, the paired Wilcoxon matched pairs signed rank test was used to determine significance.

## AUTHOR CONTRIBUTIONS

**Stephane M Guillaume:** Conceptualization; data curation; formal analysis; investigation; methodology; project administration; writing – original draft. **Helena A Carslaw:** Data curation; formal analysis; writing – review and editing. **Silvia Innocentin:** Data curation; formal analysis; investigation; methodology; writing – review and editing. **Louise M C Webb:** Conceptualization; resources; supervision; writing – review and editing. **Adrian Liston:** Resources; supervision; writing – review and editing. **William S Foster:** Supervision; writing – review and editing. **Michelle A Linterman:** Conceptualization; funding acquisition; project administration; resources; supervision; writing – original draft.

## CONFLICT OF INTEREST

MAL is a member of the GSK Immunology network and reports funding from GSK outside of this work. All other authors declare no competing financial interests.

## Supporting information


**Supplementary figure 1.** Gating strategy for human flow cytometry.
**Supplementary figure 2.** Gating strategy for mouse flow cytometry.
**Supplementary figure 3.** Activation marker expression on T_FH_ cells.

## Data Availability

Data are available from the corresponding author upon reasonable request.
