# Supplementary material for: Germinal center formation is resilient to CD69 deletion on T follicular helper cells
Source: Immunol Cell Biol. 2025 Aug 4;103(9):844–56. doi: 10.1111/imcb.70051 (PMC12521959; doi:10.1111/imcb.70051)
Supplement: Supplementary file 1 — Supplementary figure 1. Gating strategy for human flow cytometry. Supplementary figure 2. Gating strategy for mouse flow cytometry. Supplementary figure 3. Activation marker expression on TFH cells. [file IMCB-103-844-s001.pdf]

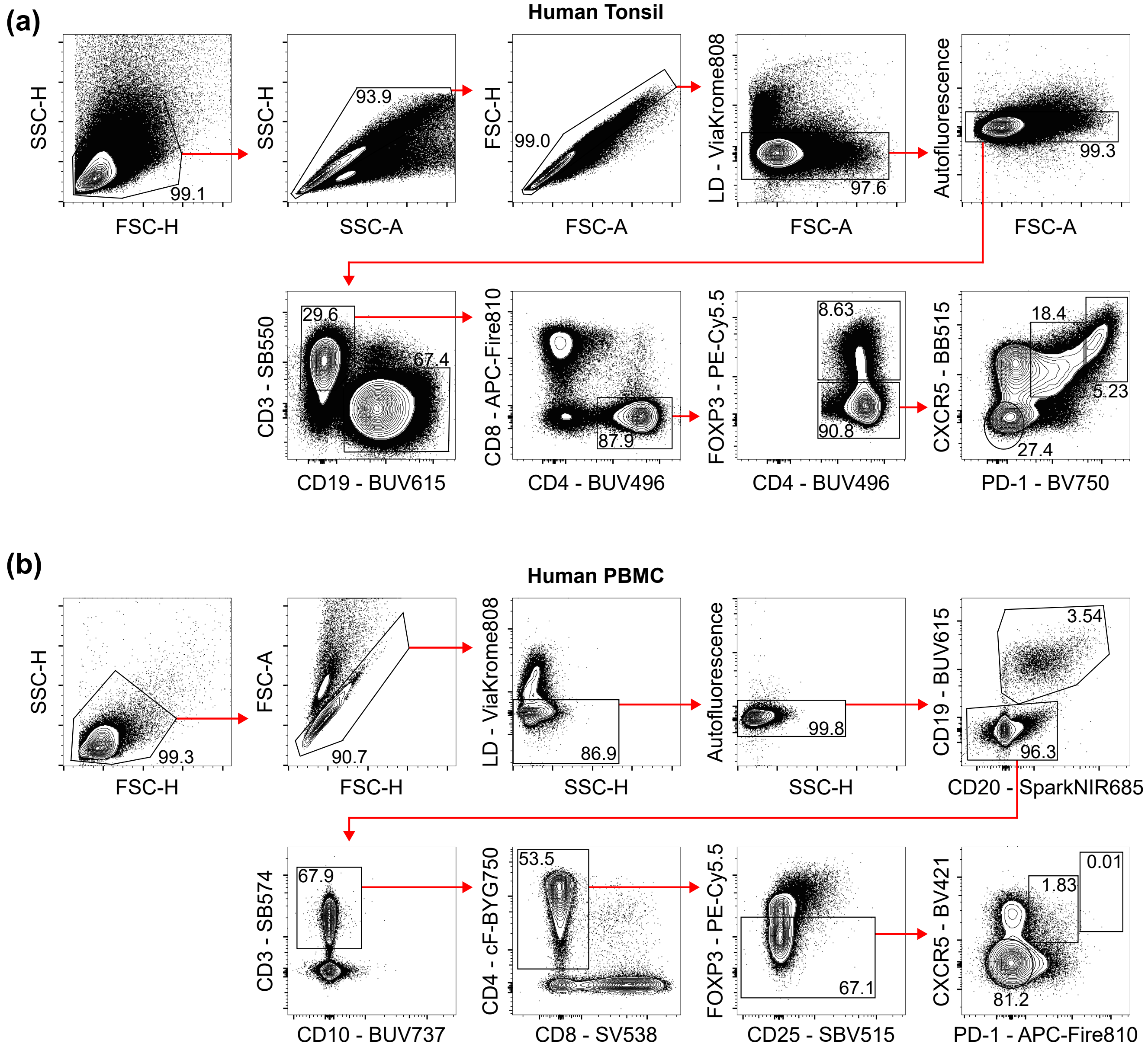

Supplementary Figure 1. Gating strategy for human flow cytometry.
(a-b) Flow cytometric gating strategy of CD4+ T cells identified in **(a)** human tonsil and **(b)** human PBMC. Contour plots are from one representative sample. Numbers in contour plots represent percentage population gated of parent population.

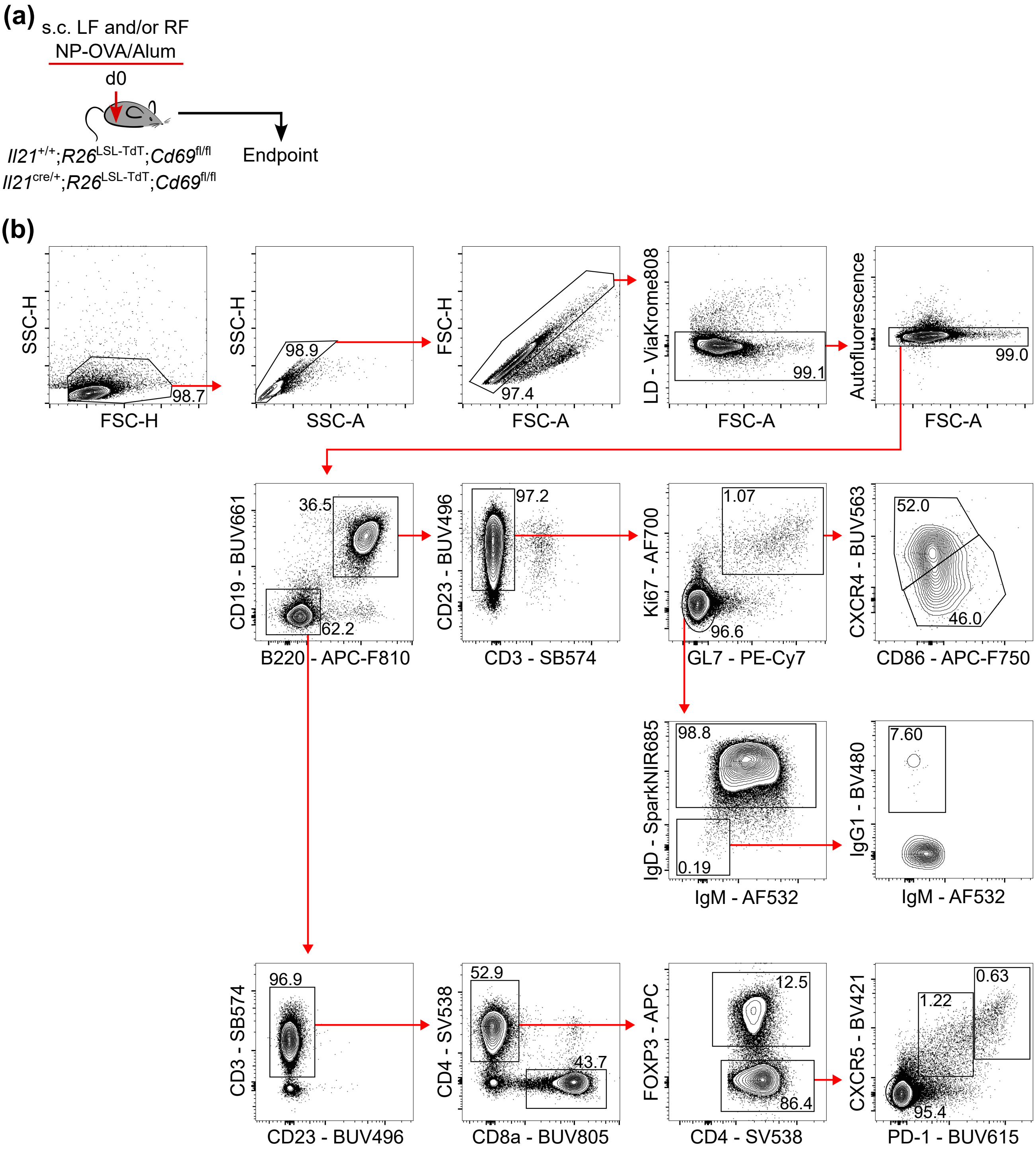

Supplementary Figure 2. Gating strategy for mouse flow cytometry.
(a) *Il21*$^{+/+}$;*R26*$^{LSL\text{-}TdT}$;*Cd69*$^{fl/fl}$ and *Il21*$^{cre/+}$;*R26*$^{LSL\text{-}TdT}$;*Cd69*$^{fl/fl}$ mice received NP-OVA/Alum s.c. in LF and/or RF and the experiment was ended at various timepoints post-immunization.
(b) Flow cytometric gating strategy of CD4^{+} T cells and B cells identified in the iLN. Contour plots are from one representative sample. Numbers in contour plots represent percentage population gated of parent population.

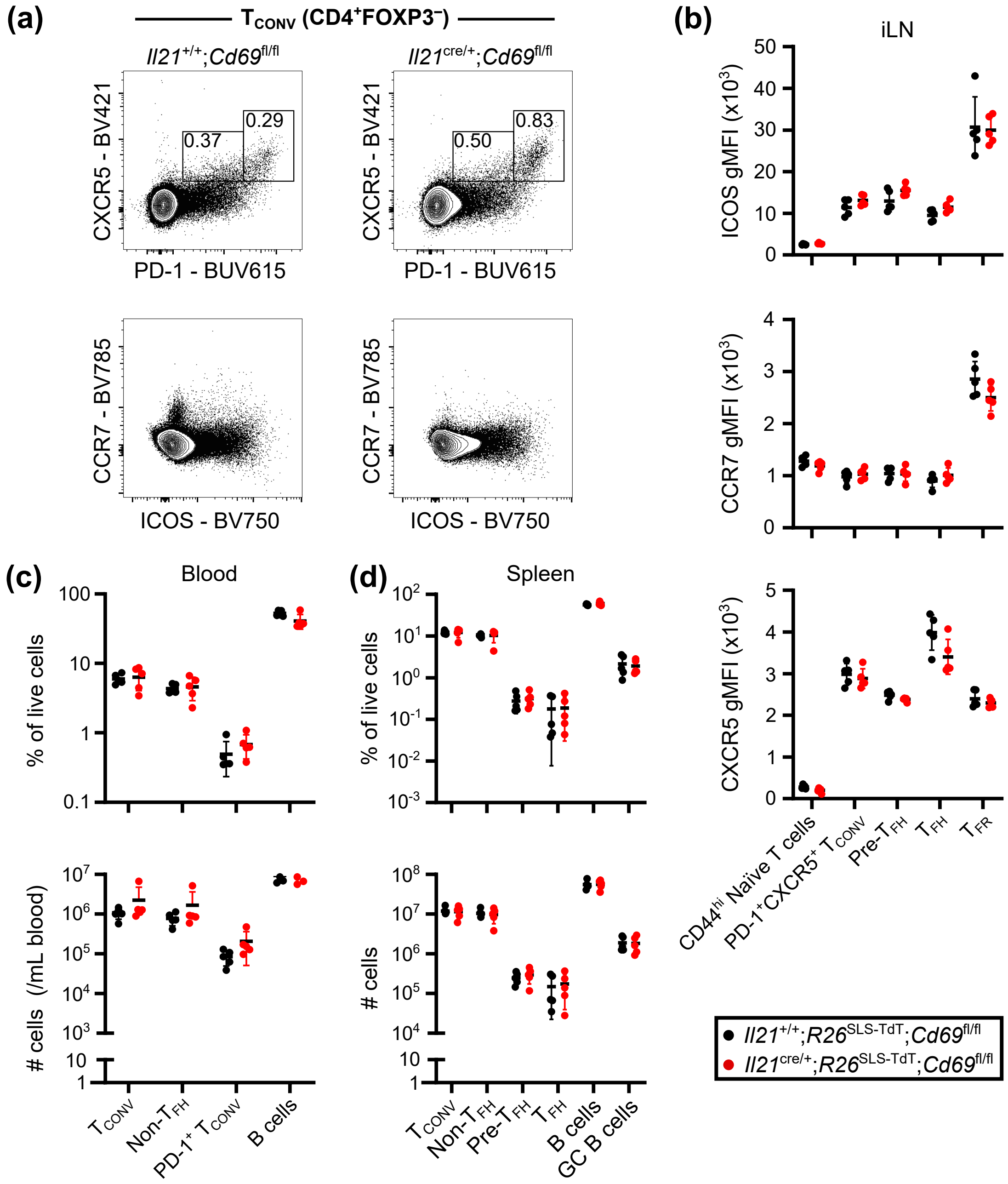

Supplementary Figure 3. Activation marker expression on T_{FH} cells.

(a) Flow cytometry contour plots of 10 dpi CD4^{+}FoxP3^{-} T_{CONV} cells from *Il21*$^{+/+}$;*R26*$^{LSL\text{-}TdT}$; *Cd69*$^{fl/fl}$ and *Il21*$^{cre/+}$;*R26*$^{LSL\text{-}TdT}$;*Cd69*$^{fl/fl}$ iLN samples. Contour plots are from one representative sample. Numbers in contour plots represent percentage population gated of parent population.

(b) gMFI of ICOS, CCR7 and CXCR5 on cells in the inguinal lymph node. Data are representative of one (CCR7) or three (CXCR5, ICOS) experiments with five mice per group.

(c) Frequency and number of cells in the blood. Data are from one experiment with five mice per group.

(d) Frequency and number of cells in the spleen. Data are representative of three experiments with five mice per group.

(b-d) Symbols on graphs indicate individual mice, lines represent mean, error bars indicate SD. Statistics used are Mann-Whitney tests with Holm-Šídák multiple corrections.
